# Exploring the Feasibility of an Electronic Tool for Predicting Retention in HIV Care: Provider Perspectives

**DOI:** 10.3390/ijerph21060671

**Published:** 2024-05-24

**Authors:** Jacqueline Kromash, Eleanor E. Friedman, Samantha A. Devlin, Jessica Schmitt, John M. Flores, Jessica P. Ridgway

**Affiliations:** 1 Pritzker School of Medicine, University of Chicago, 924 E. 57th Street, Suite 104, Chicago, IL 60637, USA; 2 Section of Infectious Diseases and Global Health, Department of Medicine, University of Chicago, 5841 S. Maryland Avenue, MC 5065, Chicago, IL 60637, USA

**Keywords:** retention in care, HIV, EHR, implementation science, providers, prescribers

## Abstract

Retention in care for people living with HIV (PLWH) is important for individual and population health. Preemptive identification of PLWH at high risk of lapsing in care may improve retention efforts. We surveyed providers at nine institutions throughout Chicago about their perspectives on using an electronic health record (EHR) tool to predict the risk of lapsing in care. Sixty-three percent (20/32) of providers reported currently assessing patients’ risk for lapsing in care, and 91% (29/32) reported willingness to implement an EHR tool. When compared to those with other job roles, prescribers agreed (vs. neutral) that the tool would be less biased than personal judgment (OR 13.33, 95% CI 1.05, 169.56). Prescribers were also more likely to identify community health workers as persons who should deliver these interventions (OR 10.50, 95% CI 1.02, 108.58). Transportation, housing, substance use, and employment information were factors that providers wanted to be included in an EHR-based tool. Social workers were significantly more likely to indicate the inclusion of employment information as important (OR 10.50, 95% CI 1.11, 98.87) when compared to other participants. Acceptability of an EHR tool was high; future research should investigate barriers and evaluate the effectiveness of such a tool.

## 1. Introduction

The HIV care continuum outlines the steps/stages that people living with HIV (PLWH) go through from diagnosis of HIV to ultimately achieving and maintaining viral suppression [1]. PLWH who receive regular medical care are more likely to be prescribed antiretroviral therapy (ART) and subsequently achieve viral suppression, resulting in a large reduction in the risk of HIV transmission [2,3]. Definitions of retention in care vary slightly, but it is generally defined as at least two attended appointments or HIV viral load tests performed at least 90 days apart within a twelve-month period [4]. However, only half of the PLWH in the U.S. are retained in care, and up to 40% are not virally suppressed [2,3,4]. Therefore, efforts to increase retention in care are essential to individual, national, and international goals of decreasing HIV-associated mortality and ending the HIV/AIDS epidemic [5].

Prior studies have identified risk factors associated with lapsing in care, which can be defined as failure to meet the above criteria for retention in care, and data show that patients have difficulty predicting their own ability to keep future appointments [6]. Common barriers to retention in care include poverty, unstable housing, mental illness, substance use disorders, transportation issues, lack of childcare, stigma, medication burden, and lack of health insurance [2,6,7,8]. Studies also show that demographic factors such as race, age, sexual orientation, and education level are associated with retention rates [2]. Patients with a history of previously lapsing in care are most likely to have future lapses in care [4,9]. Mitigating patients’ risk of lapsing in care is particularly crucial to avoid downstream medical complications that can result from not engaging in care [10]. 

Reengaging PLWH in care can be time- and resource-intensive, and it may not be feasible to implement intensive reengagement efforts for every patient who lapses in care [2]. Thus, it is crucial to preemptively identify patients who are at the highest risk of lapsing in care to effectively allocate resources [2,3,4,11,12,13]. Furthermore, it has been demonstrated that retention and re-engagement interventions can be cost-effective, especially when targeted toward high-risk groups and when used in combination with other high-impact HIV interventions [14]. Researchers have developed validated machine learning algorithms using electronic health record (EHR) data to predict the risk of lapsing in care among PLWH and to identify patients that would most benefit from retention interventions [4]. For example, a mobile application using EHR data to predict missing appointments has been shown to increase appointment attendance [13]. Predictive models of retention in care have been shown to correctly predict appointment attendance two-thirds of the time and perform adequately when applied in diverse settings [12].

What remains to be seen is how providers will interface with predictive models of retention in care. The current study aims to use implementation science methods to determine how such a model could be integrated into the workflow of HIV care teams and explores the feasibility and acceptability of using an EHR-based predictive model for identifying PLWH most at risk for lapsing in care. Accordingly, we administered a survey to healthcare clinicians and staff who work with PLWH to obtain their feedback on the utility of an electronic tool for predicting lapsing in care among PLWH.

## 2. Materials and Methods

The survey was developed using the Consolidated Framework for Implementation Research (CFIR) [15], with a focus on exploring the feasibility and acceptability of implementing the predictive model into practice to identify PLWH at risk of lapsing in care who may need additional supports. The CFIR model examines the multi-level nature of implementation across contexts and consists of five major domains: intervention characteristics (for example, HIV provider/front-line staff perceptions), outer setting (for example, barriers and facilitators for retention in care for PLWH), inner setting (for example, organizational characteristics), individual characteristics, and the process of implementation [15].

The survey included a description and example of an electronic tool that reports an individual’s likelihood of lapsing in care using an EHR-based predictive model of lapses in care among PLWH. Questions assessed staff’s confidence in the ability of the tool to accurately predict patients’ risk of lapsing in care and the likelihood that they would use the tool to guide interventions using a 5-point Likert scale. Questions also assessed staff’s confidence in the accuracy of the electronic tool to identify at-risk patients versus their own personal assessment of a patient’s risk for lapsing in care. Additionally, the survey asked which patients should be considered “at risk” and thus eligible for retention interventions (for example, patients in the highest risk quartile, patients with <50% chance of retention in the next 6 months, etc.). Lastly, the survey included questions regarding the resource implications of utilizing the tool, barriers/facilitators to using the tool, and the most feasible approach for implementing the tool within their organizational workflow. 

This study received approval from the University of Chicago Institutional Review Board (IRB21-1825). Recruitment occurred from June to December 2022. Participants were recruited via exponential, non-discriminative chain-referral sampling. We emailed a convenience sample of HIV care providers in multiple roles at clinics across Chicago, informing them of the study and inviting them to participate. The study team asked interested providers about other potential participants and/or listservs that were used for recruitment. Providers/healthcare workers were eligible to participate if they were (1) aged 18 years or older; (2) currently employed full- or part-time at a healthcare system within Chicago; (3) able to speak and understand English; and (4) able and willing to provide informed consent. No identifiable information was collected. Providers in various roles on the healthcare team (including social workers, case managers, advanced practice providers, clinic manager/directors, attending physicians, fellows, residents, health educators, etc.) self-administered the survey using the secure online platform REDCap (Research Electronic Data Capture). Providers who completed the survey received USD 50 via a mobile payment application. 

Survey analysis included descriptive statistics and exploration of sociodemographic factors associated with differences in knowledge, attitudes, and preferences about retention in care and predicting lapses in care. For Likert scale and checkbox question responses, differences between HIV care sites and the job roles of respondents were assessed. HIV care sites were grouped into categories of large academic hospitals, medium-sized hospitals, and federally qualified health centers (FQHCs). Job roles were grouped into categories of prescribers (those who can prescribe, i.e., attendings, residents/fellows, advanced practice providers), social workers (including case managers and other supportive service staff), and others (including hospital administrators or research staff). Differences in participants’ responses based on job site and job role were described using odds ratios and 95% confidence intervals (OR, 95% CI). As our outcomes of interest contained three categories, multinomial logistic regression modeling was used to produce odds ratios and confidence intervals. Likert scale responses were collapsed from six to four categories of agree/strongly agree, neutral, I don’t know or am unsure, and disagree/strongly disagree for odds ratios. Missing responses were dropped in multinomial modeling. All statistics were produced using SAS 9.4 (Cary, NC, USA).

## 3. Results

A total of 32 providers from nine healthcare organizations across Chicago completed the survey (Table 1). Most participants were white (53%, 17/32), cisgender female (69%, 22/32), and between the ages of 30 and 39 (56%, 18/32). There was a wide range of job roles represented, with attending physicians (31%, 10/32) and case managers (19%, 6/32) accounting for half of the sample. Most participants worked at the University of Chicago (53%, 17/32). 

In response to questions regarding current practices, 97% (31/32) of providers agreed that PLWH should be assessed for the risk of lapsing in care, and 63% (20/32) of providers stated that they currently do so (Table 2). However, only 34% (11/32) reported satisfaction with their organization’s current process of assessing patients’ risk of lapsing in care. The most common methods for identifying patients at high risk for lapsing in care included informal assessments (78%, 25/32), patient-initiated requests for assistance (56%, 18/32), and formal needs assessments (31%, 10/32). 

Most providers agreed (91%, 29/32) that they would utilize an electronic tool for predicting lapsing in care among PLWH (Table 2). However, only 63% (20/32) of providers are confident in the ability of the tool to accurately predict lapsing in care risk, and 50% (16/32) agreed this tool would do a better job than their personal judgments, with 53% (17/32) believing it would be less biased. Moreover, prescribers in particular agreed, as opposed to being neutral, that the tool would be less biased when compared to those with other roles (OR 13.33, 95% CI 1.05, 169.56).

The majority of providers (78%, 25/32) preferred having tool results stratified into high, medium, and low risk categories of lapsing in care (Table 2). Only 16% (5/32) of providers preferred seeing a percentage chance that a patient has for lapsing in care. Additionally, 66% (21/32) of providers would prefer to “use the tool the week before patients’ scheduled care appointments. Care coordinators would then contact high-risk patients prior to their clinic appointment to confirm and troubleshoot any potential barriers to attendance”. When asked to identify who should be responsible for providing interventions to patients at risk of lapsing in care, the most commonly identified roles included social workers (81%, 26/32) and case managers (81%, 26/32). Prescribers were significantly more likely to identify community health workers as persons who should deliver these interventions when compared to those with other job roles (OR 10.50, 95% CI 1.02, 108.58).

When asked what additional factors providers thought would be important to include in an EHR-based tool, 84% identified transportation issues (27/32), 75% identified unstable housing (24/32), and 66% identified substance use (21/32). Social workers significantly selected the inclusion of employment information as an important factor to include (OR 10.50, 95% CI 1.11, 98.87) when compared to those with other job roles (Table 2). 

Assessment of barriers to tool use showed that 56% (18/32) of providers anticipated communication issues being a barrier to use. The second most frequently anticipated barrier (50%, 16/32) was time constraints. Providers were asked to foresee the concerns that their patients might have if providers were to use the tool. The most common response was that the tool may be biased/discriminatory, with 69% (22/32) of providers predicting this to be a patient concern. The second most commonly speculated concern was that patients may be worried about privacy and confidentiality with the tool, with 59% (19/32) identifying this potential concern (Table 2).

## 4. Discussion

This study showed that across diverse healthcare roles and numerous institutions, providers in Chicago favorably viewed the potential of an EHR-based tool to help identify PLWH at high risk of lapsing in care. Providers expressed willingness to use this tool in practice while caring for PLWH and believed it would be more accurate than their current process for identifying patients at risk for lapsing in care. The results of the survey showed that this tool could potentially fill a current gap in care practices and that certain barriers would need to be addressed during the implementation process. 

We found that the majority of providers agreed that the tool should be used the week prior to a patient’s appointment and that patients with the highest risk of lapsing in care should be indicated, preferring high/medium/low risk categories over percentage risk. Broad agreement in these categories indicates the ability to have a single format that can be implemented across roles instead of needing to design specific displays. There was also consensus in the roles identified as being best suited to implement relevant interventions (i.e., social workers, community health workers, and direct service workers). It will be important to evaluate how this additional task may fit in with the existing workload of these demanding roles or what additional work this would entail to improve retention. 

Although it was not statistically significant, it is notable that mostly prescribers compared to those with other job roles identified time constraints as a barrier to tool utilization. This may be due to prescribers generally being pressed for time compared to other care team members [16], although they may not be the ones performing the retention interventions. Most of the participants who reported communication issues being a barrier worked at large hospitals, where care teams may be larger than smaller clinics [17]. This may indicate a potential need for standardized workflows on steps to be taken once a patient is flagged as high-risk of lapsing in care. In addition, although the majority of participants thought this tool would be less biased than their own personal judgment, some providers did express that patients may be concerned that the tool might be discriminatory. This suggests that work must be performed to ensure adequate provider-patient communication regarding the use of such tools. 

While EHR-based predictive tools for patient care are common, there are not currently any EHR-based tools for predicting retention in HIV care being utilized in clinical practice. Wohlfeiler et al. conducted implementation science research to evaluate outcomes in clinics using an app that integrates some EHR data, such as appointment history, to identify PLWH who may miss upcoming appointments and send out daily alerts to providers. They found that clinics using the app had higher rates of appointment attendance for patients that were flagged as at-risk for lapsing in care than control clinics [13]. Studies of this kind can be used in tandem with the provider perspectives described here to inform the implementation of a predictive model that does not require the installation of a separate app or program but instead has the potential to be integrated into the EHR system that providers are already using on a regular basis. Additionally, the prevalence of predictive reports in the EHR system reasonably indicates that a tool of this kind can be introduced into care practices, as we have seen for similar tools that predict sepsis events, hospital readmissions, and other events, which have improved patient outcomes [18].

Our study is among the first to investigate provider perspectives on using an EHR predictive tool for retention in HIV care. Van den Berg et al. previously conducted 10 focus groups among primary care providers to assess perspectives on a pre-exposure prophylaxis (PrEP) candidate prediction tool among patients without HIV and found that providers are receptive to using such a tool [19]. These providers expressed similar concerns as those in our study regarding potential patient concerns about accuracy and ethics, supporting future work in assessing patient willingness to use a tool for HIV retention risk assessment. Surveys of provider acceptability of the previously described mobile application for identifying patients with HIV likely to miss upcoming appointments found that providers wanted the system to be built directly into the EHR [13]. Our study adds to this prior work by providing additional perspectives on the best ways to utilize an EHR-based prediction model for lapsing in care among PLWH. 

Although the sample population was not large, participants in our study came from different backgrounds and care settings, suggesting that our results may be applicable to larger populations. Provider opinions were not uniform. Both the diversity of demographics and responses support generalizability to similar settings (i.e., HIV clinics in large, urban cities). Future studies on the use of this EHR-based tool could verify and expand the findings of this small sample. A potential limitation of our study is that participants were not randomly sampled but instead used exponential, non-discriminative chain-referral sampling. As a result, we did not have a way of tracking the overall response rate, as we cannot estimate how many HIV care providers are in Chicago or how many people saw our study recruitment materials or were referred to the study by previous participants. Of the providers who responded to the recruitment email expressing interest in participating, 100% completed the survey. It is possible that of all those who received the recruitment email, people who more favorably viewed the tool may have been more likely to fill out the survey, so our results may be skewed by this participation bias. Additionally, we only surveyed providers in the Chicago area, and a majority of respondents were from our home institution, the University of Chicago. Because HIV care may not function similarly in other settings (for example, smaller cities with different demographics and more rural areas), further work is needed to evaluate how this tool may function in other locations. It will also be important for future studies to qualitatively examine the perspectives and potential concerns amongst patients with regard to this tool, as our study only surveyed providers. In this manuscript, we largely present descriptive results due to our limited sample size. Future studies with larger sample sizes should be used to further explore comparative analyses. 

## 5. Conclusions

We found that providers believed a tool for predicting PLWH at risk of falling out of care would be effective and were willing to use such a tool. This study was an important first step in identifying barriers to tool implementation and provides a path for future work in relieving those barriers. Although this study focused on provider perspectives, the evaluation of patient perspectives on this tool will be important to assess prior to implementation. 

## Figures and Tables

**Table 1:** Participant demographics.

Variable	*n* (N = 32)	Percent (%)
**Age (years)**		
18–29	2	6
30–39	18	56
40–49	8	25
50–59	2	6
60 or older	2	6
**Gender**		
Cisgender Female	22	69
Cisgender Male	10	31
**Race**		
Asian/Asian American/Pacific Islander	5	16
Black/African American	5	16
Middle Eastern/Arab American	3	9
Native American/American Indian	1	3
Two or more races	1	3
White	17	53
**Ethnicity**		
Hispanic/Latino	3	9
Not Hispanic/Latino	28	88
Missing	1	3
**Workplace**		
University of Chicago	17	53
Howard Brown Health	1	3
Sinai Health	6	19
Rush University	3	9
Loyola Health	1	3
Chicago Family Health Center	1	3
Greater Family Health	1	3
South Side Help Center	1	3
Northwestern Medicine	1	3
**Job Title**		
Social Worker	3	9
Case Manager	6	19
Advanced Practice Manager	2	6
Clinic Manager/Director	2	6
Attending Physician	10	31
Fellow/Resident	3	9
Health Educator	2	6
Mental Health Specialist	1	3
Research Nurse	1	3
Linkage to Care Coordinator	1	3
Project Coordinator	1	3

**Table 2:** Select survey responses.

Statement/Question	*n* (N = 32)	Percent (%)
**Patients with HIV should be assessed for risk of lapsing in HIV care.**		
Agree/Strongly Agree	31	97
Neutral	1	3
Disagree/Strongly Disagree	0	0
I don’t know/unsure	0	0
**I routinely assess my patients for their risk of lapsing in care.**		
Agree/Strongly Agree	20	63
Neutral	5	16
Disagree/Strongly Disagree	7	22
I don’t know/unsure	0	0
**I am satisfied with our clinic** **’s process for identifying patients at risk of lapsing in care.**		
Agree/Strongly Agree		
Neutral	11	34
Disagree/Strongly Disagree	10	31
I don’t know/unsure	9	28
Missing	1	3
	1	3
**How do you currently identify patients who are at risk of lapsing in care and who may require additional support to attend their appointments? ^1^**		
Nothing	0	0
Informal Assessment	25	78
Formal Needs Assessment	10	31
Patient Initiates request for assistance	18	56
Other	3	9
I don’t know	1	3
**I would use the tool to guide interventions for patients at risk of lapsing in care.**		
Agree/Strongly Agree	29	91
Neutral	2	6
Disagree/Strongly Disagree	0	0
I don’t know/unsure	1	3
**I feel confident in the ability of an electronic tool to accurately predict clients** **’ risk of lapsing in care.**		
Agree/Strongly Agree	20	63
Neutral	8	25
Disagree/Strongly Disagree	2	6
I don’t know/unsure	2	6
**I think the electronic tool would be more accurate and better at identifying patients at risk of lapsing in care than the current system or my own judgment.**		
Agree/Strongly Agree		
Neutral	16	50
Disagree/Strongly Disagree	8	25
I don’t know/unsure	4	13
Missing	3	9
	1	3
**I think the electronic tool may be less biased than the current system or my own judgment.**		
Agree/Strongly Agree		
Neutral	17	53
Disagree/Strongly Disagree	10	31
I don’t know/unsure	2	6
Missing	2	6
	1	3
**How would you want to see the results of the tool regarding patients at risk of lapsing in care?**		
High/medium/low risk for lapsing in care	25	78
Percentage chance a patient has for lapsing in care	5	16
Other (both and unsure)	2	6
**If an electronic tool for identifying patients’ risk of lapsing in care were accessible in your clinic, what would be the most useful way to utilize it?**		
Use the tool every morning prior to the start of clinic, to produce a list of patients scheduled for a clinic appointment that day as well as their likelihood of lapsing in care	0	0
Use the tool the week before patients’ scheduled care appointments. Care coordinators would then contact high-risk clients prior to their clinic appointment to confirm and troubleshoot any potential barriers to attendance	21	66
At time of making appointment	1	3
Have the tool available on-demand for providers to use at any time.	5	16
I don’t know/unsure	3	9
Other: more than one of these,	2	6
pop up when you open a patient chart, make it available to the MAs and other support staff		
**Who should be responsible for providing interventions to help clients at risk of lapsing in care attend their clinic appointments? ^1^**		
Social workers	26	81
Case managers	26	81
Health educators	14	44
Patient service representatives	14	44
Schedulers	13	41
Nurses	12	38
Physicians or advanced practice providers	11	34
Community health workers	13	41
Other	1	3
I don’t know/I am unsure	1	3
**The current electronic tool uses EHR data to predict risk of lapsing in care. Data include previous missed appointments, age, gender, race, diagnoses, lab results, and other demographic factors. If you were to design a tool to predict risk of lapsing in care, what other information about the patient would you want to include in the tool? ^1^**		
Unstable housing	24	75
Transportation issues	27	84
Mental health	20	63
Substance use	21	66
Lapse in insurance	18	56
Criminal justice involvement	9	28
Employment issues (unemployed, employed but working off-hours/shifts that make it difficult to attend appointments, etc.)	14	44
Childcare/elder care/caretaker responsibilities	16	50
Other: Cognitive impairment, presence of an advocate in the home, social support	3	9
I don’t know/I am unsure	2	6
None of these	0	0
**Assuming your clinic had access to the electronic tool, which of the following are barriers that might prevent you from using it? ^1^**		
Unnecessary for patient care	1	3
Time constraints	16	50
Low comfort level with interpreting the results of the tool	3	9
Communication issues (i.e., ensuring the proper staff member receives the results of the electronic tool and intervenes accordingly)	18	56
Already too many prompts/charting templates in the EHR	8	25
Do not have resources to intervene to assist clients identified as at risk of lapsing in care	9	28
Do not believe the results of the tool are accurate	2	6
Concerns about the ethics of a computerized system making predictions about patients based on their electronic medical record data	7	22
Other	2	6
**What concerns do you think patients would have with the electronic tool? ^1^**		
Privacy/confidentiality	19	59
The tool is inaccurate	17	53
The tool is biased/discriminatory	22	69
The tool is irrelevant/unnecessary for them	13	41
Providers will not give them the resources they need if they use the tool	9	28

^1^ Multiple responses allowed.

## Data Availability

The raw data supporting the conclusions of this article will be made available by the authors on request.
